# Pinoresinol Diglucoside Alleviates oxLDL-Induced Dysfunction in Human Umbilical Vein Endothelial Cells

**DOI:** 10.1155/2016/3124519

**Published:** 2016-11-30

**Authors:** Jinpeng Yao, Zhipeng Zou, Xiangfen Wang, Xiaoping Ji, Jun Yang

**Affiliations:** ^1^Department of Cardiology, Qilu Hospital of Shandong University, Jinan, Shandong 250012, China; ^2^Department of Cardiology, Yantai Yeda Hospital of Binzhou Medical University, Yantai, Shandong 264100, China; ^3^Department of Geriatrics, Yantai Yeda Hospital of Binzhou Medical University, Yantai, Shandong 264100, China; ^4^Yantai Yuhuangding Hospital of Qingdao University, Yantai, Shandong 264000, China

## Abstract

Atherosclerotic cardiovascular diseases are the leading causes of morbidity and mortality worldwide. Deposition of oxidized low-density lipoprotein (oxLDL) is one of the initiators and promoters of atherosclerosis.* Eucommia* lignans were shown to possess antihypertensive effects. This study aimed to investigate the effects of pinoresinol diglucoside (PD), a* Eucommia* lignan, on oxLDL-induced endothelial dysfunction. HUVECs were treated with oxLDL and/or PD followed by assessing radical oxygen species (ROS), apoptosis, nitrogen oxide (NO), malondialdehyde (MDA), and superoxide dismutase (SOD) activity with specific assays kits, mRNA levels with quantitative real-time polymerase chain reaction (PCR), and protein levels with western blot. PD abolished oxLDL-induced ROS and MDA production, apoptosis, upregulation of lectin-like oxidized LDL recptor-1 (LOX-1), intercellular Adhesion Molecule 1 (ICAM-1), and nuclear factor kappa-light-chain-enhancer of activated B-cells (NF-*κ*B), and activation of p38MAPK (mitogen-activated protein kinases)/NF-*κ*B signaling. Meanwhile, PD alleviated oxLDL-caused inhibition of SOD activity, eNOS expression, and NO production. These data demonstrated that PD was effective in protecting endothelial cells from oxLDL-caused injuries, which guarantees further investigation on the clinical benefits of PD on cardiovascular diseases.

## 1. Introduction

Cardiovascular diseases (CVDs) are the leading causes of death and burden of disease worldwide [1, 2]. As the underlying cause of most CVDs including stroke and myocardial infarction, atherosclerosis is considered as a chronic inflammatory disease which is initiated by endothelial injuries and progressed with the deposition of oxidized low-density lipoprotein (oxLDL) and inflammatory cells (monocytes, macrophages, etc.) to the arterial wall [3–5]. Though many pathways are involved in the development of atherosclerosis, oxLDL plays a critical role throughout the process by promoting the recruitment of inflammatory cells, increasing endothelium injuries, stimulating the proliferation of vascular smooth muscle cells, and promoting the production and release of inflammatory mediators including radical oxygen species (ROS) and cytokine [3–6]. Besides, oxLDL promotes monocyte adhesion to endothelial cells through ROS and NF-*κ*B activation [6–8].

OxLDL-induced oxidative stress and ROS production play a critical role in endothelial injury and [9]. OxLDL could stimulate NADPH oxidase production of ROS by activating AMPK/PKC pathway [10] or increasing mitochondrial ROS generation [11]. The chronic high level ROS causes inflammation and endothelial injury, which are the deciding processes for the initiation and progression of atherosclerosis.

Pinoresinol diglucoside (PD) is one of the major lignans isolated from* Eucommia ulmoides *Oliver bark which is called Duzhong in Traditional Chinese medicine. Lignans, iridoids, flavonoids, polysaccharides, terpenes, and proteins have been identified from* Eucommia* bark [12].* Eucommia* extract was shown to possess antioxidative effect [12, 13], hypoglycemic and hypolipidemic effects [14, 15], and antihypertensive effect [16, 17]. Lignans, the major bioactive compounds of* Eucommia*, was shown to inhibit hypertensive renal injury [18] and angiotensin II induced proliferation [19, 20] and extracellular matrix production [20] in rat mesangial cells. This study aimed to investigate whether PD has protective effects against oxLDL-induced endothelial dysfunction.

## 2. Methods

### 2.1. Cell Culture

Human umbilical vein endothelia cells (HUVECs) were purchased from ATCC (Manassas, VA) and maintained in DMEM (SH30022.01B, Hyclone, Logan, UT) supplemented with 10% fetal bovine serum (16000-044, Life Technologies, Shanghai, China), 0.1 mg/mL heparin (Life Technologies), 50 *μ*g/mL endothelial cell growth supplement (E2759, Sigma, St. Louis, MO), and 100 U/mL penicillin and 100 *μ*g/mL streptomycin (Hyclone) in a humidified incubator with 5% CO_2_ at 37°C.

### 2.2. LDL Isolation and Oxidation

All protocols were approved by the institute review committee of Shandong University. A written informed consent form was obtained from each healthy blood donor. LDL was isolated from plasma of healthy individuals and oxidized according to previously described methods [21–23]. Briefly, native LDL was isolated by sequential ultracentrifugation (1.019 < *d* < 1.063 g/mL) of plasma in the presence of EDTA and dialyzed against 500 volumes of PBS for 48 h at 4°C to remove the EDTA. LDL was oxidized by incubating LDL in 0.5 mg/mL protein with 5 *μ*mol/L CuSO_4_ for 18 h. The amount of thiobarbituric acid reactive substances was monitored using a TBARS assay kit (10009055, Cayman Chemical, Ann Arbor, MA) according to manufacturer's instructions and the values of malondialdehyde (MDA) equivalents increased from 0.93 ± 0.16 nmol/mg protein of native LDL to 21.7 ± 2.3 nmol/mg protein of CuSO_4_-treated LDL. Then, oxLDL samples were dialyzed against 500 volumes of PBS at 4°C for 48 h to remove the Cu^2+^ and were filtered through a 0.45 *μ*m filter. The protein concentrations of native LDL and oxLDL were measured with a Pierce™ Coomassie Plus (Bradford) Assay Kit (23236, ThermoFisher, Grand Island, NY).

### 2.3. Pinoresinol Diglucoside (PD)

PD was purchased from Qingdao Jie Shi Kang Biotechnology (Qingdao, China), which had a purity ≥ 99%. PD was purified from* Eucommia ulmoides *Oliver bark. The CAS number of PD is 63902-38-5, and molecular formula is C_32_H_42_O_16_ with a molecular weight of 682.668 g/mol. PD stock solution was 10 mmol/L in methanol and working solution was diluted with culture medium.

### 2.4. Apoptosis Assay

HUVECs were seeded into 6-well plates (2 × 10^5^/well) and cultured for 24 hrs before experimental treatments. The cells were pretreated with 0.1 or 1 *μ*mol/L of pinoresinol diglucoside (PD) for 60 min where indicated before being treated with or without 100 *μ*g/mL of oxLDL for 24 hrs. The cells were then collected and stained using FITC Annexin V Apoptosis Detection Kit with PI (640914, Biolegend, San Diego, CA) according to the manufacturer's protocol. Briefly, the cells were washed twice with cold BioLegend's Cell Staining Buffer and resuspended in Annexin V Binding Buffer at a concentration of 0.5–1.0 × 10^7^ cells/mL. Then, 100 *μ*L of cell suspension was transferred into a 5 mL test tube, mixed with 5 *μ*L of FITC Annexin V and 10 *μ*L of Propidium Iodide Solution, and incubated at room temperature in the dark for 15 min with gentle vortexing. 400 *μ*L of Annexin V Binding Buffer was added to each tube before being analyzed on a BD Accuri™ C6 (BD Biosciences, Shanghai, China).

### 2.5. ROS Assessment

The levels of intracellular ROS were analyzed using OxiSelect™ Intracellular ROS Assay Kit (STA-342, Cell Biolabs, San Diego, CA) after HUVECs were treated with 100 *μ*g/mL of oxLDL and/or 1 *μ*mol/L of PD (60 min prior to adding oxLDL) for 24 hrs. Briefly, the cells were washed 3 times with DPBS after oxLDL/PD treatment and 200 *μ*L of 1x DCFH-DA (2′,7′-dichlorodihydrofluorescin diacetate) media solution was added to the cells, which was incubated at 37°C for 30 min always in light. The cells were then washed 3 times with DPBS and analyzed on a BD Accuri C6 or photographed on an Olympus IX70 Inverted Microscope (Olympus, Shanghai China).

### 2.6. Measurement of NO

The intracellular NO content was assessed with Total Nitric Oxide Assay Kit (S0023, Beyotime, Shanghai, China) following manufacturer's instructions. After 24 hrs treatment with oxLDL and/or PD, the HUVECs were lyzed with Cell and Tissue Lysis Buffer for Nitric Oxide Assay (S3090, Beyotime) and centrifuged at 14000 ×g at 4°C for 10 min. 60 *μ*L of supernatant was transferred into 96-well assay plate, mixed with 5 *μ*L of NADPH (2 mmol/L), 10 *μ*L of FAD, and 5 *μ*L of nitrate reductase, and incubated 30 min at 37°C. LDH Buffer and LDH (10 *μ*L each) were added into assay mixture followed by 30 min incubation at 37°C. Then, 50 *μ*L of Griess Reagent I and 50 *μ*L Griess Reagent II were added and the reaction was continued at room temperature for 10 min before the absorbance was measured at 540 nm on a microplate reader (BioTek, Beijing, China).

### 2.7. Assessment of MDA Content

The MDA content was measured using a TBARS assay kit (10009055, Cayman Chemical) according to manufacturer's protocol. Briefly, HUVECs after oxLDL and/or PD treatment for 24 hrs were lyzed in PBST (10^6^ cells in 0.1 mL PBST) by sonication on ice. 100 *μ*L of cell lysate or standard was added to a 5 mL vial, which was mixed in 100 *μ*L of SDS solution and 4 mL of the color reagent. The vials were capped and boiled for 60 min, incubated on ice for 10 min, and centrifuged 10 min at 1600 ×g at 4°C. 150 *μ*L of supernatant was transferred into an assay plate and read on a FLx800 fluorescent reader (BioTek) with excitation 530 nm and emission 550 nm.

### 2.8. Analysis of Cellular Total Superoxide Dismutase (SOD) Activity

The total SOD activity was measured using a Total Superoxide Dismutase Assay Kit with NBT (S0109, Beyotime) following the manufacturer's protocol. After 24 hrs treatment with oxLDL and/or PD, HUVECs were washed twice with cold PBS, lyzed in PBS by pulse sonication on ice, and centrifuged at 13000 ×g at 4°C for 10 min. The supernatant was transferred into fresh tube. The sample protein concentration was measured with Pierce Coomassie Plus (Bradford) Assay Kit (23236, ThermoFisher) and adjusted to 1 *μ*g/*μ*L. 20 *μ*L of sample or SOD assay buffer (blanks) was added to 96-well assay plate, 160 *μ*L NBT/enzyme working solution was added into all wells, and 20 *μ*L of reaction initiation working solution was added to all wells except for Blank2. The plate was incubated at 37°C for 30 min before being read at 560 nm on a microplate reader (BioTek). The SOD activity was calculated as follows:

(1) $$\begin{array}{c} \%\text{ inhibition}=\frac{\left(A\text{blank}1-A\text{sample}\right)}{\left(A\text{blank}1-A\text{blank}2\right)}*100. \end{array}$$

SOD enzyme activity in the sample = %  inhibition/(1 − %  inhibition).

### 2.9. Quantitative Real-Time PCR

The total RNA from HUVECs treated with oxLDL and/or PD for 24 hrs was extracted with Trizol reagent (ET111-01, TransGen Biotech, Beijing, China). The first-strand cDNA was synthesized using an M-MLV Reverse Transcriptase kit (Invitrogen, Carlsbad, CA) and was diluted 10-fold with RNase free ddH_2_O before use. Real-time quantitative RT-PCR was executed with an SYBR Premix Ex TaqTM kit (Takara, Dalian, China) in a 20 *μ*L reaction volume on a ABI Step One (Applied Biosystems, Foster City, CA) with the following cycling program: 95°C for 2 min followed by 40 cycles of 95°C for 15 sec, 58°C for 30 sec, and 68°C for 30 sec. The relative mRNA level was calculated using the 2^−ΔΔCt^ method with GAPDH as the internal control. The primers were listed in Table 1.

### 2.10. Western Blot

The HUVECs were lyzed in 100 *μ*L of RIPA buffer (50 mM Tris-cl pH 7.4, 150 mM NaCl, 1% NP40, and 0.25% Na-deoxycholate) containing 1x complete protease inhibitor cocktail (Roche Diagnostics, Indianapolis, IN) and 1x phosphatase inhibitor cocktail (Sigma, St. Louis, MO) on ice for 30 min. After centrifugation at 10000 ×g at 4°C for 15 min, the supernatant was collected and protein concentration was measured with Pierce Coomassie Plus (Bradford) Assay Kit (23236, ThermoFisher). 30 *μ*g total protein was resolved in a 8% sodium dodecyl sulfate polyacrylamide gel and transferred onto a PVDF membrane, which was blocked in 5% nonfat milk in PBST (0.1% Tween 20 in PBS) at room temperature for 30 min followed by being incubated with specified first antibodies at 4°C overnight. Next day, the membranes were washed 3 times at 5 min each time with PBST and then incubated with proper horseradish peroxidase-conjugated secondary antibodies (Jackson ImmunoResearch, West Grove, PA) for 60 min at room temperature. The specific bands were detected with Pierce ECL Plus Substrate (ThermoFisher). The primary antibodies used were p-NF-*κ*B p65 (ab86299), NF-*κ*B p65 (ab16502), ICAM-1 (ab124759), and LOX-1 (ab60178) from Abcam (Cambridge, MA), p-p38MAPK (4511), and p38MAPK (8690) from Cell Signaling (Shanghai, China) and *β*-actin (TA-09) from ZSGB-Bio (Beijing, China).

### 2.11. Statistical Analysis

All experiments were performed in triplicate independently for at least three times. The data was presented as mean ± SEM. The differences between groups were analyzed using one-way analysis of variance or Student's *t*-test. It was considered statistically significant if *p* < 0.05.

## 3. Results

### 3.1. Pinoresinol Diglucoside Inhibits oxLDL-Induced Endothelial Apoptosis

**Figure 1:**
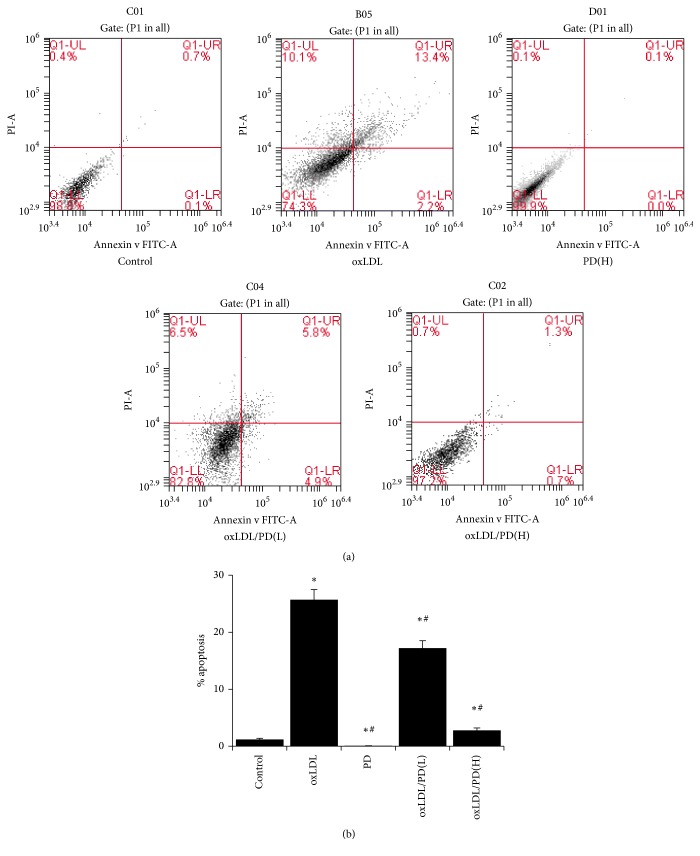
Pinoresinol diglucoside inhibits oxLDL-induced apoptosis. (a) The apoptosis of HUVECs was analyzed by Annexin V/PI staining followed by flow cytometry. HUVECs were cultured for 24 hrs without treatment, with 100 *μ*g/mL of oxLDL, 1 *μ*mol/L of PD, 100 *μ*g/mL of oxLDL plus 0.1 *μ*mol/L of PD, and 100 *μ*g/mL of oxLDL plus 1 *μ*mol/L of PD. (b) Quantitative analysis of percentage of apoptotic cells of HUVECs with the different treatment listed in (a). Experiments were performed 3 independent times with 3 replications. ^*∗*^ *p* < 0.05 compared to control cells; ^#^ *p* < 0.05 compared to oxLDL-treated cells.

Treatment of 100 *μ*g oxLDL for 24 hrs caused 25.7% HUVECs undergoing apoptosis, which was significantly higher than that of control cells (1.2%) (Figure 1). Pretreating cells with 0.1 *μ*mol/L of PD reduced the apoptotic cells to 17.2% whereas 1 *μ*mol/L of PD abrogated the apoptosis-inducing effect of oxLDL (Figure 1). Therefore, the 1 *μ*mol/L dose of PD was used in ensuing experiments.

### 3.2. Pinoresinol Diglucoside Alleviates Oxidative Stress Caused by oxLDL

**Figure 2:**
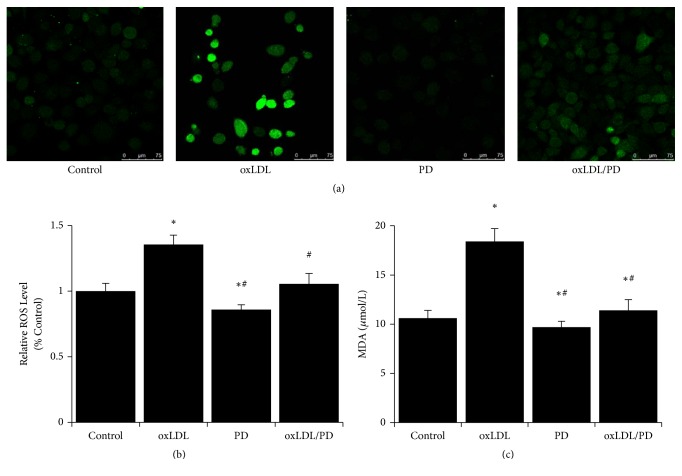
Pinoresinol diglucoside alleviates oxidative stress caused by oxLDL. (a) Representative pictures of HUVECs stained with DCFH-DA after being cultured without treatment, with 100 *μ*g/mL of oxLDL, 1 *μ*mol/L of PD, and 100 *μ*g/mL of oxLDL plus 1 *μ*mol/L of PD for 24 hrs. (b) The relative ROS levels of HUVECs were analyzed by flow cytometry following DCFH-DA staining after being cultured without treatment, with 40 *μ*g/mL of oxLDL, 1 *μ*mol/L of PD, and 40 *μ*g/mL of oxLDL plus 1 *μ*mol/L of PD for 24 hrs. (c) The lipid peroxidation was assessed by measuring the contents of malondialdehyde in HUVECs treated with oxLDL and/or PD. Experiments were performed 3 independent times with 3 replications. ^*∗*^ *p* < 0.05 compared to control cells; ^#^ *p* < 0.05 compared to oxLDL-treated cells.

The ROS levels in HUVECs were assessed using DCFH-DA staining followed by flow cytometry or fluorescence microscopy. The production of ROS in HUVECs treated with oxLDL was markedly increased compared to control cells (Figures 2(a) and 2(b)). The presence of PD reduced the ROS level by more than 20% in oxLDL-treated HUVECs (Figures 2(a) and 2(b)). Meanwhile, the level of lipid peroxidation (MDA content) increased more than 70% by oxLDL treatment and about 80% of the increased MDA level was inhibited by PD (Figure 2(c)).

### 3.3. Pinoresinol Diglucoside Reverses the oxLDL-Induced Reduction of SOD Activities

**Figure 3:**
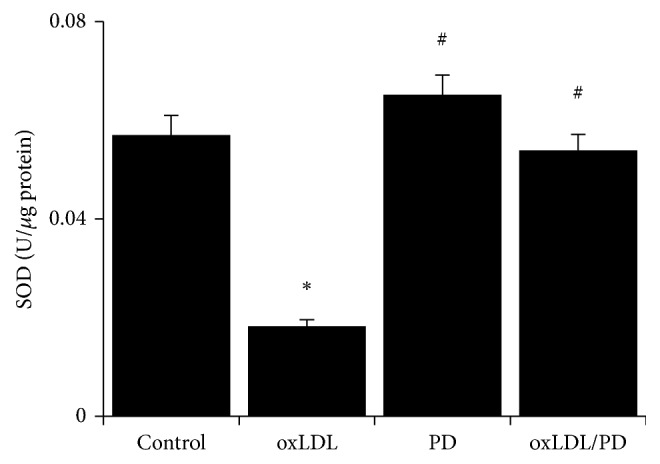
Pinoresinol diglucoside relieves the inhibition of SOD activity by oxLDL. The total SOD enzymatic activity of HUVECs treated with 40 *μ*g/mL oxLDL and/or 1 *μ*mol/L PD was analyzed using a commercially available assay kit. Experiments were performed 3 independent times with 3 replications. ^*∗*^ *p* < 0.05 compared to control cells; ^#^ *p* < 0.05 compared to oxLDL-treated cells.

The total SOD activities of oxLDL-treated HUVECs were reduced about 68% compared to control cells (Figure 3). Pretreating HUVECs with pinoresinol diglucoside abrogated the inhibition of SOD activities by oxLDL (Figure 3).

### 3.4. Pinoresinol Diglucoside Alleviates oxLDL-Induced Inhibition of NO Production in HUVECs

**Figure 4:**
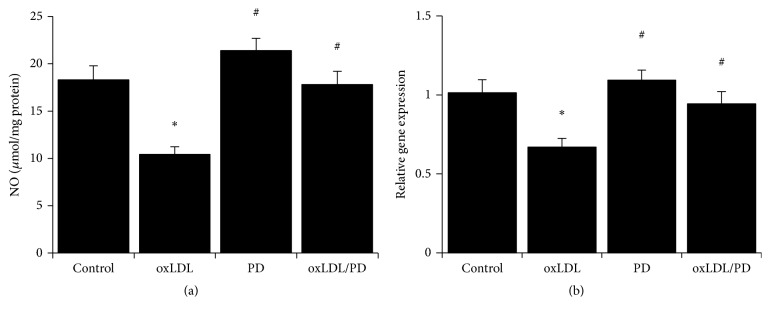
Pinoresinol diglucoside alleviates oxLDL-induced inhibition of NO production. (a) The total NO metabolites levels in HUVECs treated with 40 *μ*g/mL oxLDL and/or 1 *μ*mol/L PD were measured using a commercial assay kit. (b) The mRNA levels of eNOS in HUVECs treated with 40 *μ*g/mL oxLDL and/or 1 *μ*mol/L PD were assessed by quantitative real-time PCR. Experiments were performed 3 independent times with 3 replications. ^*∗*^ *p* < 0.05 compared to control cells; ^#^ *p* < 0.05 compared to oxLDL-treated cells.

The HUVECs treated with oxLDL produced more than 40% less NO compared to untreated cells (Figure 4(a)). This decreased production of NO in oxLDL-treated HUVECs was fully inhibited by pinoresinol diglucoside (Figure 4(a)). Correspondingly, oxLDL-caused over 30% reduction of the expression of eNOS compared to untreated HUVECs (Figure 4(b)). Pretreatment of HUVECs with PD completely abolished the inhibition of eNOS expression by oxLDL (Figure 4(b)).

### 3.5. Pinoresinol Diglucoside Inhibits the Expression of Genes Involved in Inflammation, Adhesion, and oxLDL Uptake

**Figure 5:**
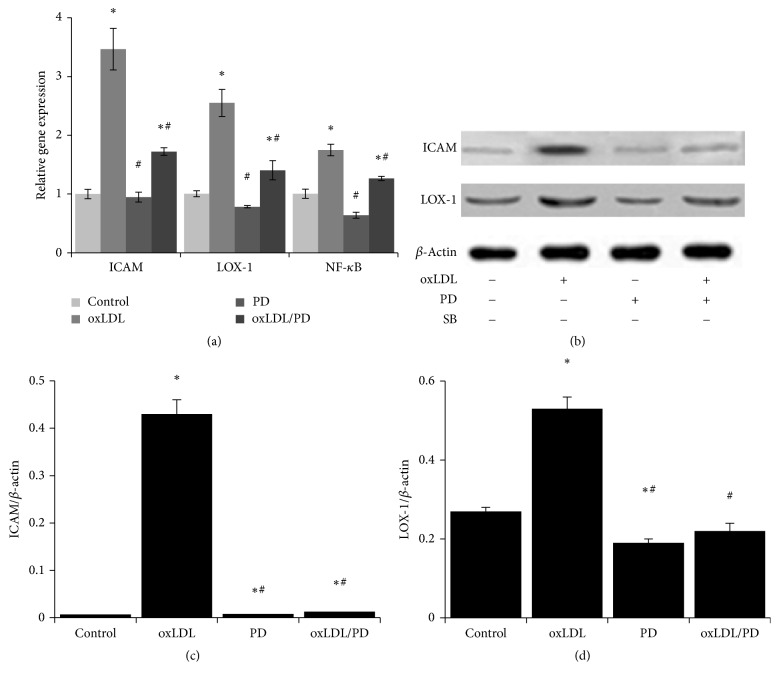
Pinoresinol diglucoside inhibits oxLDL-induced increase of LOX-1, ICAM, and NF-*κ*B expression. (a) The mRNA levels of LOX-1, ICAM1, and NF-*κ*B were analyzed using quantitative real-time PCR. (b) The protein levels of LOX-1 and ICAM were evaluated by western blot. The results of quantitative analysis of ICAM (c) and LOX-1 (d) western blot were shown. Experiments were performed 3 independent times with 3 replications. ^*∗*^ *p* < 0.05 compared to control cells; ^#^ *p* < 0.05 compared to oxLDL-treated cells.

The mRNA (Figure 5(a)) and protein (Figures 5(b)–5(d)) levels of lectin-like oxidized low-density lipoprotein receptor-1 (Lox-1), intercellular adhesion molecule 1 (CAM1), and nuclear factor of kappa light polypeptide gene enhancer in B-cells 1 (NF-*κ*B) in HUVECs were upregulated 1.7- to 3.5-fold by oxLDL, and such upregulation was essentially abrogated by pinoresinol diglucoside.

### 3.6. The oxLDL Promoted Activation of p38MAPK/NF-*κ*B Signaling Is Inhibited by Pinoresinol Diglucoside

**Figure 6:**
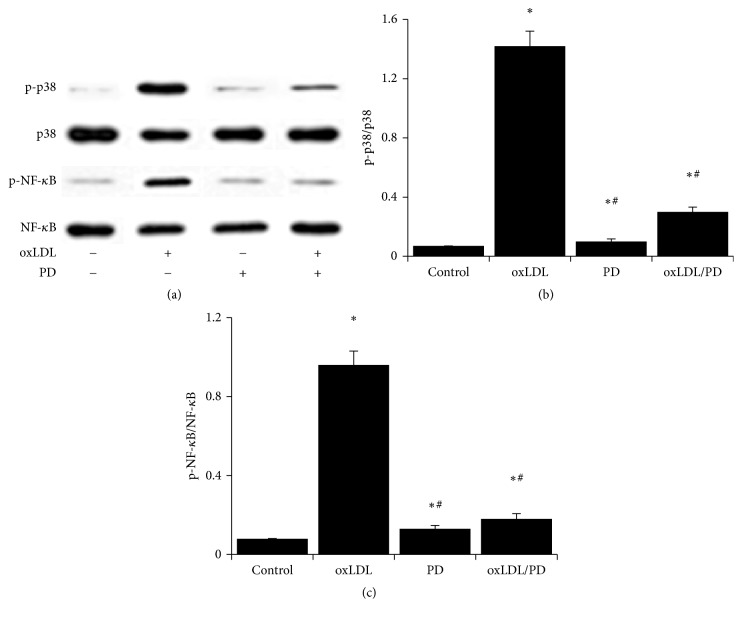
The oxLDL promoted activation of p38MAPK/NF-*κ*B signaling is inhibited by pinoresinol diglucoside. (a) HUVECs were treated with or without 1 *μ*mol/L of PD for 60 min before being treated with 40 mg/mL of oxLDL for 45 min. The levels of p-p38MAPK, p38MAPK, p-NF-*κ*B p65, and NF-*κ*B p65 were analyzed by western blot. The ratio of p-p38MAPK/p38MAPK (b) and p-NF-*κ*B p65/NF-*κ*B p65 (c) was shown. Experiments were performed 3 independent times with 3 replications. ^*∗*^ *p* < 0.05 compared to control cells; ^#^ *p* < 0.05 compared to oxLDL-treated cells.

Treating HUVECs with oxLDL-induced strong phosphorylation of p38MAPK and NF-*κ*B p65 (Figure 6). Pinoresinol diglucoside alone did not have obvious effects on p38MAPK or NF-*κ*B p65 phosphorylation but it significantly inhibited oxLDL-induced phosphorylation of p38MAPK and NF-*κ*B p65 (Figure 6).

## 4. Discussion

Pinoresinol diglucoside, a natural flavanone found in licorice, showed the ability to protect endothelial cells from oxLDL-induced damages. Specifically, pinoresinol diglucoside alleviated cellular oxidative stress and endothelial apoptosis caused by oxLDL, reversed the inhibition of NO production by oxLDL, and inhibited oxLDL-induced overexpression of genes involved in cell adhesion, inflammation, and oxLDL uptake. Moreover, pinoresinol diglucoside effectively inhibited the activation of p38MAPK and NF-*κ*B by oxLDL.

Oxidized LDL has long been shown to cause the death of endothelial cells through excessive oxidative stress [21, 23, 24], and the oxLDL-caused endothelial dysfunction could be intervened by targeting different aspects of oxLDL actions. Isothiocyanates sulforaphane, benzyl isothiocyanate, and phenethyl isocyanate promoted the expression of heme oxygenase-1 and glutamate cysteine ligase in a nuclear factor erythroid 2-related factor 2 (Nrf2) dependent manner, resulting in subdued ROS production and NF-*κ*B activation in HUVECs upon oxLDL treatment [23]. Another study showed that thioredoxin inhibited oxLDL-induced expression of endothelial cell adhesion molecules (ICAM and VCAM) in HUVECs by promoting the phosphorylation, nuclear translocation, and nuclear retention of SMAD3 protein [25]. The expression of T-box 20 gene was shown to be suppressed by oxLDL in HUVECs or the arteries of mice fed with high-fat diet; and overexpressing T-box 20 reduced oxLDL-induced ROS production and expression of intercellular adhesion molecules in HUVECs through promoting peroxisome proliferator-activated receptors gamma expression [26]. These data demonstrated that the excessive ROS production and overexpression of cell adhesion molecules were the main theme of oxLDL-induced endothelial dysfunction.

**Figure 7:**
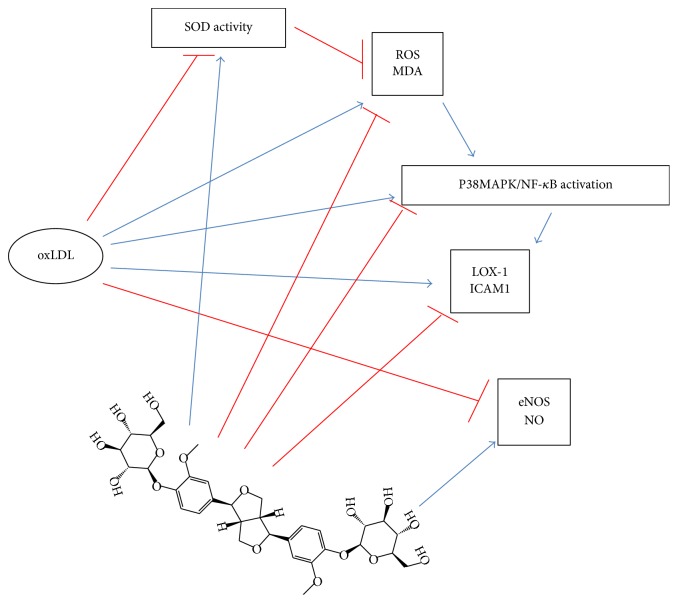
The proposed working model for pinoresinol diglucoside protecting HUVECs against oxLDL-induced injuries. Refer to test for details.

Additionally, oxLDL has been shown to induce the expression and the dissociation from microtubules of endothelial arginase II as well as its translocation from mitochondria into cytoplasm to reduce the levels of endothelial nitric oxide, resulting in the impairment of NO signaling and related endothelial function [27, 28]. The activation of arginase II by oxLDL was through a LOX-1 dependent RhoA/ROCK pathway [29], which was consistent with the observation that arginase II was dissociated from microtubules by oxLDL [28]. Phosphocreatine antagonized the apoptotic effect of oxLDL by activating PI3K/Akt/eNOS pathway and NO production in HUVECs [30]. Meanwhile, p38MAPK/NF-*κ*B pathway has been shown to be strongly activated by oxLDL and served as an effective target for intervention [31, 32]. Moreover, puerarin protected against oxLDL-caused endothelial injury by inhibiting oxLDL-induced LOX-1 overexpression, p38MAPK activation, and eNOS inhibition [32]. Our data demonstrated that pinoresinol diglucoside exerted multifaceted effects in protecting HUVECs against oxLDL-induced endothelial dysfunction (Figure 7). PD blocked the activation of p38MAPK/NF-*κ*B and LOX-1 signaling to diminish the acute effects of oxLDL and would prevent endothelial adhesion of inflammatory cells. PD stimulated NO production and chronically inhibition of ROS production induced by oxLDL would maintain proper endothelial functions.

## 5. Conclusion

PD inhibited oxLDL-induced upregulation of LOX-1 and ICAM-1, ROS production, lipid peroxidation, and p38MAPK/NF-*κ*B activation. Meanwhile, PD also relieved the inhibition of SOD activity, NO production, and eNOS expression by oxLDL in HUVECs, indicating that PD was effective in protecting endothelial cells from oxLDL-caused injuries and warranting further studies on its potential clinical benefits in treating cardiovascular diseases.

## Figures and Tables

**Table 1:** PCR primer sequence.

Primer	Sequence
GAPDH_F	CCACTAGGCGCTCACTGTTC
GAPDH_R	AGGCGCCCAATACGACCAA
hNF-kB_F	GCAGATGGCCCATACCTTCA
hNF-kB_R	TAGAGGCACCAGGTAGTCCA
hICAM1_F	CCCCTCAAAAGTCATCCTGC
hICAM1_R	GGGTCTCTATGCCCAACAACT
hLox-1-F	CTGACCTCCTAACACAAG
hLox-1-R	TGAAGTCCAGATCAGCTC
heNOS-F	GATGAGTATGACGTGGTGTCCC
heNOS-R	CCGAGGGGAGCTGTTGTA
